# Dietary habits and stroke: A Mendelian randomization analysis

**DOI:** 10.1097/MD.0000000000048588

**Published:** 2026-05-08

**Authors:** Fei He, Na Li, Shangling Xie, Jie Yang, Zhen Wang, Yixuan Li, Si Yuan

**Affiliations:** a College of Acupuncture, Massage and Rehabilitation, Hunan University of Chinese Medicine, Changsha, Hunan, China; b South China Research Center for Acupuncture and Moxibustion, Medical College of Acu-Moxi and Rehabilitation, Guangzhou University of Chinese Medicine, Guangzhou, Guangdong Province, China.

**Keywords:** cardiovascular and cerebrovascular health, dietary habits, Mendelian randomization, single-nucleotide polymorphisms, stroke

## Abstract

Stroke is one of the diseases with a high incidence rate in the world, which endangers human health. Existing studies suggest an association between diet and stroke, but whether there is a causal relationship has not been clarified. With 9 common dietary habits as exposures and stroke as the outcome, we explored the causal relationship between dried fruit intake and stroke. We used 5 Mendelian randomization methods and 3 methods for sensitivity analysis. The 3 dietary habits obtained with the inverse-variance weighted method were associated with stroke. The results are as follows: alcohol usually taken with meals (odds ratio [OR] = 0.57, 95% confidence interval [CI] = 0.37–0.79, *P* = .007), dried fruit intake (OR = 0.51, 95% CI = 0.37–0.70, *P* = 3.69 × 10^−5^), and cheese intake (OR = 0.73, 95% CI = 0.58–0.92, *P* = .002). The relationship between dietary alcohol and cheese intake, and the finding that dried fruit intake can reduce the risk of stroke, have significant implications for the prevention and management of stroke.

## 1. Introduction

Stroke is a prevalent and increasingly common disease worldwide. Annually, approximately 795,000 individuals experience a new or recurrent stroke. Of these, approximately 610,000 represent initial attacks, while 185,000 are recurrent attacks (Greater Cincinnati/Northern Kentucky Stroke Study, National Institute of Neurological Disorders and Stroke, and National Heart, Lung, and Blood Institute; Greater Cincinnati/Northern Kentucky Stroke Study and National Institute of Neurological Disorders and Stroke data for 1999 provided on July 9, 2008; unpublished estimates compiled by the National Heart, Lung, and Blood Institute).^[1]^ Furthermore, the absolute burden of stroke is projected to increase over the next 3 decades in the majority of EU countries, particularly in eastern states. In light of the estimated 27% increase in the number of people surviving a stroke in Europe, coupled with a reduction in the proportion of individuals of working age, there is a pressing need to intensify efforts to prevent stroke.^[2]^

Diet is often an essential means of preventing disease. A substantial body of evidence from epidemiological studies indicates that dietary factors are associated with cardiovascular disease (CVD), including heart disease, stroke, and type 2 diabetes.^[3–5]^ A reduction in sodium intake, avoidance of egg yolks, a limitation of the intake of animal flesh (particularly red meat), and an increase in the intake of whole grains, fruits, vegetables, and lentils would contribute significantly to the reversal of the trend toward increased cardiovascular risk in China.^[6]^ These findings indicate that diet is closely linked with stroke. In addition, people’s dietary habits have a significant impact on different diseases. Many diseases are often closely related to more than a dozen everyday dietary habits.^[7–11]^ Building on the Global Dietary Guidelines as a framework,^[12]^ we screened a large number of dietary habits that are often used as exposure factors in observational studies and are supported by reliable data in genomic databases. Among them, 9 dietary habits have the most significant impact on stroke risk, including salt added to food, bread intake, grains intake, cheese intake, fresh fruit intake, dried fruit intake, oily fish intake, non-oily fish intake, and alcohol usually taken with meals.^[13–15]^ In primary prevention, dietary modifications are a crucial strategy for reducing stroke risk and maintaining human health. However, the current study examined only the association between diet and stroke; whether there is a causal relationship remains unknown. In addition, there is a wide variety of diets. Conducting randomized clinical trials takes significant time and cost, and the effects of confounding factors and reverse causation cannot be excluded in observational studies.

Mendelian randomization (MR) is an analytical approach for assessing the causal interpretation of an observed association between a modifiable exposure or risk factor and a clinically relevant outcome.^[16]^ It is a valuable tool, particularly when randomized controlled trials to test causality are not feasible and observational studies provide biased associations due to confounding or reverse causality.^[17]^ In an MR analysis, genetic variants, usually single-nucleotide polymorphisms (SNPs), are instrumental variables (IVs) for the putative risk factor. The principle of MR refers to Mendel’s second law of independent segregation of genetic alleles as deoxyribonucleic acid is transmitted from parents to offspring during gamete formation. This is similar to random treatment allocation in a randomized controlled trial, which aims to create groups with similar clinical characteristics to reduce the risk of confounding.^[18]^ There are many MRs published on stroke^[19–21]^ and MR articles on the causal relationships between diet, sedentary lifestyles, and other diseases.^[22–24]^

This study aimed to investigate whether 9 common dietary habits are causally related to stroke and to identify dietary patterns that are causally related to stroke. This will be an essential factor in reducing the incidence of stroke in the future, and it will also be significantly helpful in the dietary care of stroke patients.

## 2. Methods

### 2.1. Study design

**Figure 1.**
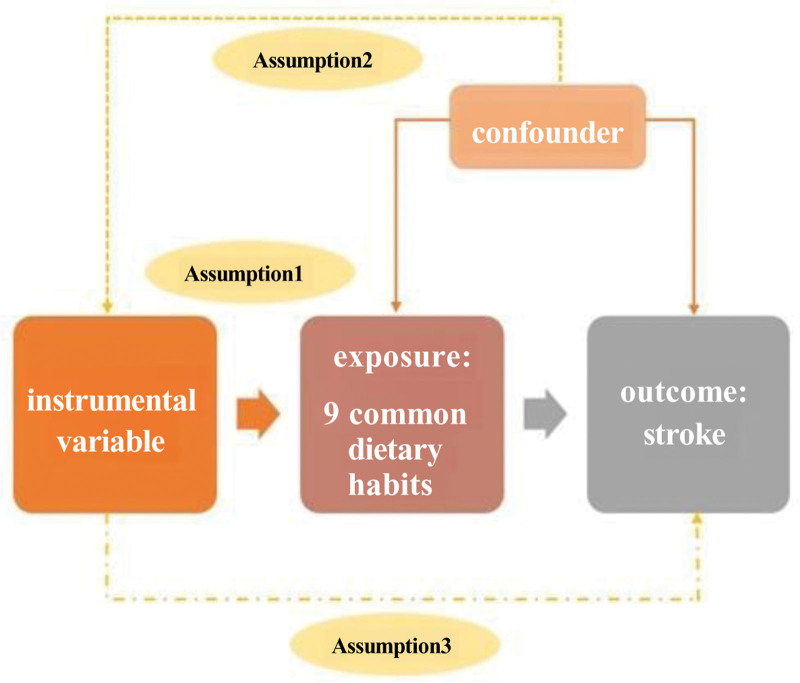
The basic principles of the MR study show the 3 principal assumptions. MR = Mendelian randomization.

We used a two-sample MR design to investigate the causal relationship between diet and stroke. The 3 core assumptions that must be met for valid results in an MR analysis are illustrated in Figure 1; specifically, the genetic variant (or multiple genetic variants) used as the IV for the risk factor must be reliably associated with the risk factor under investigation (relevance assumption); not be associated with any known or unknown confounders (independence assumption); and influence the outcome only through the risk factor and not through any direct causal pathway (exclusion restriction assumption).^[18]^

### 2.2. Data source

In this research, the 9 genetic tools for dietary habits were derived from the IEU OpenGWAS project (https://gwas.mrcieu.ac.uk/), a database of 346,664,978,019 genetic associations from 50,044 genome-wide association study (GWAS) summary datasets that can be queried or downloaded. The 9 dietary habits include salt added to food, bread intake, grains intake, cheese intake, fresh fruit intake, dried fruit intake, oily fish intake, non-oily fish intake, and alcohol usually taken with meals. Comprehensive information on the dietary habits datasets is presented in Table 1; full details are available from the original public websites cited. Data on stroke (GWAS ID: GCST006906) from the GWAS Catalog (https://www.ebi.ac.uk/gwas/), a database of human GWASs, with summary statistics, diagrams, ancestry information, and more. Among these, the total sample size of stroke was 446,696 cases, including 40,585 cases of European descent and 406,111 controls of European descent. A total of 8,255,860 SNPs were recorded.

**Table 1:** Detailed information on GWAS (genome-wide association study) summary statistics for the 9 dietary habits.

Exposures	GWAS ID	Sample size	Number of SNPs	Population	Sex
Salt added to food	ukb-b-8121	462,630	9,851,867	European	Males and females
Bread intake	ukb-b-11348	452,236	9,851,867	European	Males and females
Cereal intake	ukb-b-15926	441,640	9,851,867	European	Males and females
Cheese intake	ukb-b-1489	451,486	9,851,867	European	Males and females
Fresh fruit intake	ukb-b-3881	446,462	9,851,867	European	Males and females
Dried fruit intake	ukb-b-16576	421,764	9,851,867	European	Males and females
Oily fish intake	ukb-b-2209	460,443	9,851,867	European	Males and females
Non-oily fish intake	ukb-b-17627	460,880	9,851,867	European	Males and females
Alcohol usually taken with meals	ukb-b-16878	235,645	9,851,867	European	Males and females

SNP = single-nucleotide polymorphism.

### 2.3. Selection of IVs

To pick the SNP most strongly associated with diet, we set *P* < 5 × 10^−8^ as the criterion, and we performed linkage disequilibrium (LD) analysis with *R*^2^ < 0.001 and a cluster distance of 10,000 kb. Moreover, MR is used to test and estimate the magnitude of a causal effect of a phenotype on an outcome using genetic variants as IVs. The association estimates from IV analyses are biased by observed confounding between the phenotype and outcome. The magnitude of the bias depends on the *F*-statistic (IV strength) for the association between the IVs and the phenotype.^[25]^ In this study, we used the formula to calculate the *F* value as *F* = β^2^/SE^2,[26]^ where β is the effect size of the allele and SE is the standard error, an essential measure of the accuracy of the effect value estimate. Therefore, we removed the SNPs with an *F* value < 10 to further ensure the SNP’s strong association with the diet.

### 2.4. MR analysis

The R package “Two Sample MR” (MRC Integrative Epidemiology Unit, University of Bristol, Bristol, United Kingdom; http://github.com.hnucm.opac.vip/MRCIEU/TwoSampleMR), currently curated by MR-Base, was used to perform MR analysis of the data.^[27]^ Our study adopted the default 5 MR methods in this R package for the analysis: inverse-variance weighted (IVW), weighted median, weighted mode, simple mode, and Mendelian randomization Egger regression (MR-Egger).

1. The most efficient analysis with valid IVs is the IVW method with multiplicative random effects. It is considered the primary analysis method for pooled data, accounting for heterogeneity in variant-specific causal estimates, but its 0-intercept constraint makes it susceptible to pleiotropy.^[28]^
2. The MR-Egger method can assess whether genetic variation has pleiotropic effects (directional pleiotropy) and provides a consistent estimate of the instrument number strongly influences degree of estimation bias (independent of causal effect) under weaker assumptions. However, suppose the instrument number strongly influences degree of estimation bias hypothesis is violated (i.e., the pleiotropic effect of the genetic variant in the analysis is unrelated to its association with risk factors). In that case, the peripheral variant’s impact on the MR-Egger estimate is profound, undermining the causal inference of the MR-Egger method.^[29]^
3. The weighted median combines the data from several genetic variants into a single estimate of causality. This estimator is consistent with up to 50% invalid IV information and complements the proposed MR-Egger method.^[30]^
4. Weighted mode: combines MR estimates by weighting the effects of different genetic variants, using the mode of these results to minimize outlier bias.^[31]^
5. Simple mode: an unweighted method utilizing empirical density for effect estimation.^[31]^

### 2.5. Sensitivity analysis

The Cochran *Q* statistic can be used to quantify heterogeneity.^[32]^ In MR studies with large summary data samples, the between-instrument *Q* test effectively assesses heterogeneity arising from pleiotropy or other factors.^[33]^ Directional pleiotropy can be tested using MR-Egger.^[34]^ MR-Egger provides a valid test of directional (unbalanced) pleiotropy and of the causal null hypothesis, assuming that the slope estimated from the MR-Egger is a consistent estimate of the actual causal effect.^[35]^ The intercept term represents the estimate of directional pleiotropic effects.^[36]^ In addition, we used the “leave-one-out” technique to remove a particular SNP to observe whether the remaining SNPs, as IVs, significantly impacted the entire outcome. We present this result visually using forest plots, showing that the results remain robust, with no significant change in any SNPs.

### 2.6. Ethical review

This manuscript does not include clinical studies or patient data. All data are sourced from publicly available databases, thus avoiding ethical controversy. Therefore, no additional ethical approval or informed consent is required.

## 3. Results

### 3.1. Selection of IVs

This study examined the causal relationship between 9 common dietary habits and stroke, using stroke as the outcome. A total of 35 SNPs were considered as genetic IVs for stroke based on alcohol, usually taken with meals, as an exposure factor. A total of 43 SNPs are closely associated with stroke, with dried fruit intake as the exposure factor. A total of 64 SNPs were identified as genetic tools for stroke based on cheese intake as an exposure factor. While using salt added to food, bread intake, cereal intake, fresh fruit intake, oily fish intake, non-oily fish intake as exposure factors, there were 102, 31, 43, 54, 61, and 10 genetic IVs that may be associated with stroke, respectively. Before the final screening yielded SNPs, we removed data with *P* > 5 × 10^−8^ and performed further screening with LD < 0.001 to remove LD. When the screening process was completed, we finally calculated the *F* values of alcohol, usually taken with meals, dried fruit intake, and cheese intake, the 3 strongly correlated exposure factors, and found that the *F* values were all between 29.74 and 137.32, indicating that the weak IVs had little impact on the screening results. Finally, the required SNPs were obtained. Specific information on the final screening SNP is available in Table S1, Supplemental Digital Content.

### 3.2. MR estimations

**Figure 2.**
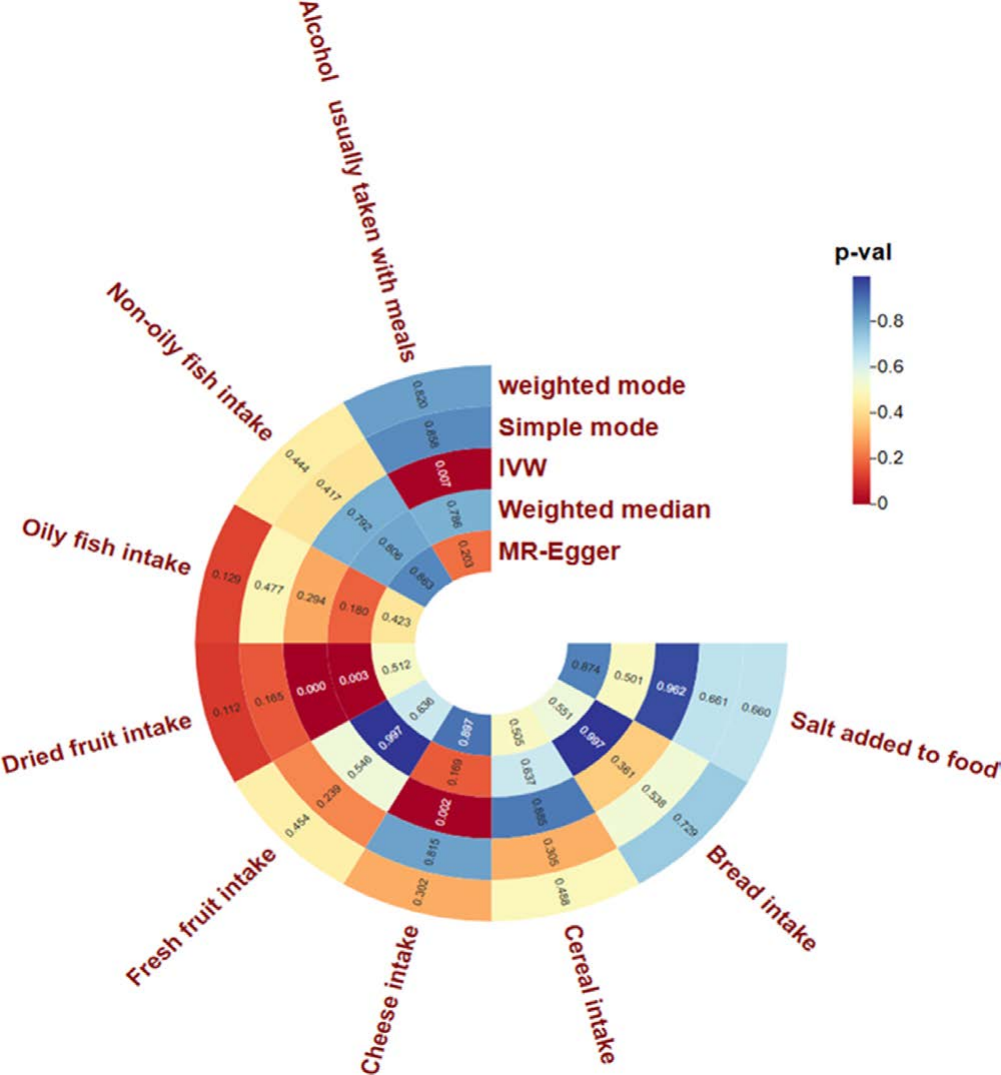
The circular heat map was used to show the MR results displayed in 5 methods. The closer to the blue *P*-value is, the greater the correlation, indicating that this diet is not associated with stroke. The closer to the red *P*-value is, the smaller the correlation, focusing on the result of *P* < .05, indicating that this diet is more strongly associated with stroke. The specific value in the space represents the corresponding *P*-value. IVW = inverse-variance weighted, MR-Egger = Mendelian randomization Egger regression.

**Figure 3.**
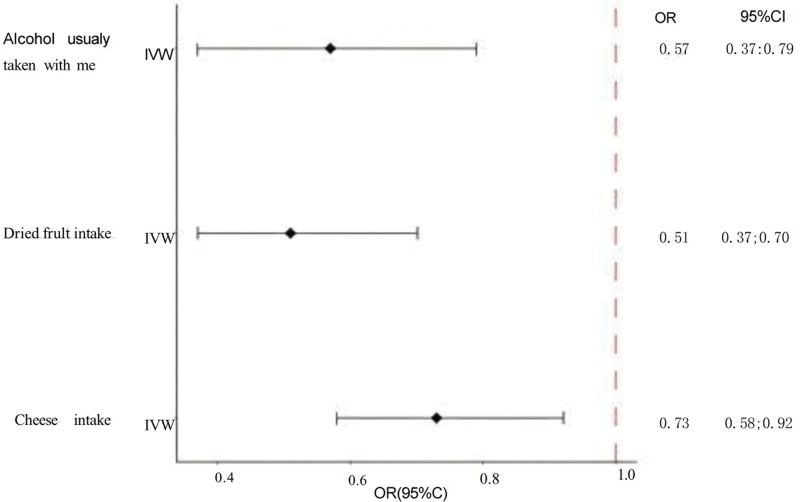
The IVW method showed 3 dietary habits with significant results. In the diagram, the line segment represents the CI, and the solid point represents the OR. CI = confidence interval, IVW = inverse-variance weighted, OR = odds ratio.

**Figure 4.**
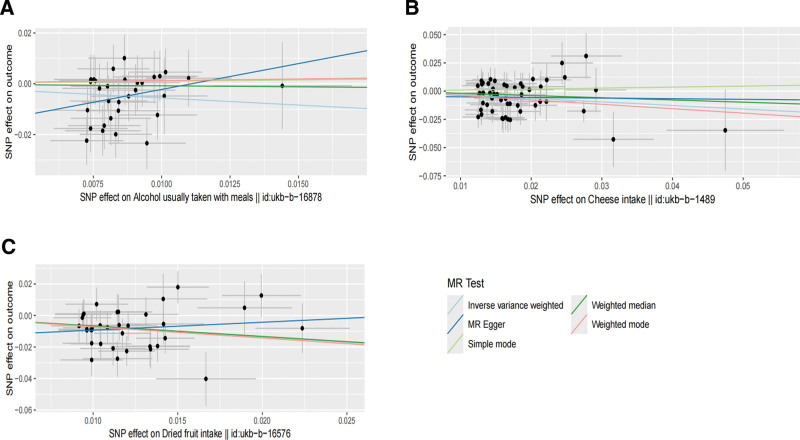
A scatter plot further showed the MR analysis results. (A) Represents the scatter plot with alcohol usually taken with meals as the exposure and stroke as the outcome. (B) Represents a scatter plot with cheese intake as the exposure and stroke as an outcome. (C) Represents a scatter plot with dried fruit intake as exposure and stroke as an outcome. The slope of the line represents beta values, and if the slope is <0, it means the exposure reduces beta < 0, the risk of the outcome, and vice versa. MR = Mendelian randomization, MR-Egger = Mendelian randomization Egger regression, SNP = single-nucleotide polymorphism.

We used 5 methods to explore the causal relationship between diet and stroke, and first presented the final MR results as a circular heat map. The circular heat map analysis, focusing on IVW *P* < .05 results, clearly indicates that 3 of 9 dietary habits are strongly associated with stroke risk. These 3 nutritional habits are associated with alcohol usually taken with meals, dried fruit intake, and cheese intake. The ring heat map is derived from *ChiPlot* (Jianmin Xie, Beijing, China; https://www.chiplot.online). Specific information is shown in Figure 2. Because IVW is usually considered the most precise and stable in determining causality,^[37]^ we refined the results using forest plots in Figure 3. We found that these 3 dietary habits’ odds ratio (OR) and confidence interval (CI) values were located on the left side of the dotted line, which may have played an essential role in reducing stroke risk. According to the IVW study, we found that alcohol usually taken with meals (OR = 0.57, 95% CI = 0.37–0.79, *P* = .007), dried fruit intake (OR = 0.51, 95% CI = 0.37–0.70, *P* = 3.69 × 10^−5^), cheese intake (OR = 0.73, 95% CI = 0.58–0.92, *P* = .002). Using the weighted median, we found that dried fruit intake was the most significant result: dried fruit intake (OR = 0.52, 95% CI = 0.33–0.80, *P* = .002). This result further validates the strong correlation between dried fruit intake and stroke. We also present the MR results using the 5 methods with scatter plots (Fig. 4A–C). We found that the slopes (beta values) of the IVW and weighted median methods were negative in Figure 4A, indicating that the MR results of these 2 methods are consistent, that is, the alcohol usually taken with meals was negatively associated with stroke. In Figure 4B, we used cheese intake as exposure. We found that the 4 slopes other than the simple mode were negative, indicating a greater likelihood that cheese intake is negatively associated with stroke. In Figure 4C, when dried fruit intake was used as the exposure, the 4 methods, except MR-Egger, found a slope <0 and a steep slope, again indicating the robustness of the negative correlation between dried fruit intake and stroke. Specific data on 3 dietary habits that are clearly associated, and the 5 methods can be queried in Table S2, Supplemental Digital Content.

### 3.3. Sensitivity analysis

To further test the reliability of the MR results, we have performed appropriate sensitivity analyses. We found no heterogeneity in the results using the Cochran *Q* test. Both the MR-Egger and IVW methods yielded *P*-values > .05, indicating no significant heterogeneity and further verifying the reliability of this experiment. Table 2 contains the specific results of the heterogeneity tests.

**Table 2:** Cochran *Q* test was used to analyze sensitivity.

Exposure	Outcome	Methods	*Q*-value	*Q*-df	*Q*-pval
Alcohol usually taken with meals	Stroke	MR-Egger	24.967	29	0.680
		Inverse-variance weighted	27.765	30	0.583
Dried fruit intake	Stroke	MR-Egger	38.384	33	0.239
		Inverse-variance weighted	41.344	34	0.181
Cheese intake	Stroke	MR-Egger	63.602	51	0.111
		Inverse-variance weighted	64.086	52	0.121

*Q*-value and *Q*-df (*Q* statistic degrees of freedom) are essential statistics to assess consistency between study results. While *Q*-values reflect the degree of inter-study variation, *Q*-df provides information about the degrees of freedom needed to determine if this variation is statistically significant.

MR-Egger = Mendelian randomization Egger regression.

**Figure 5.**
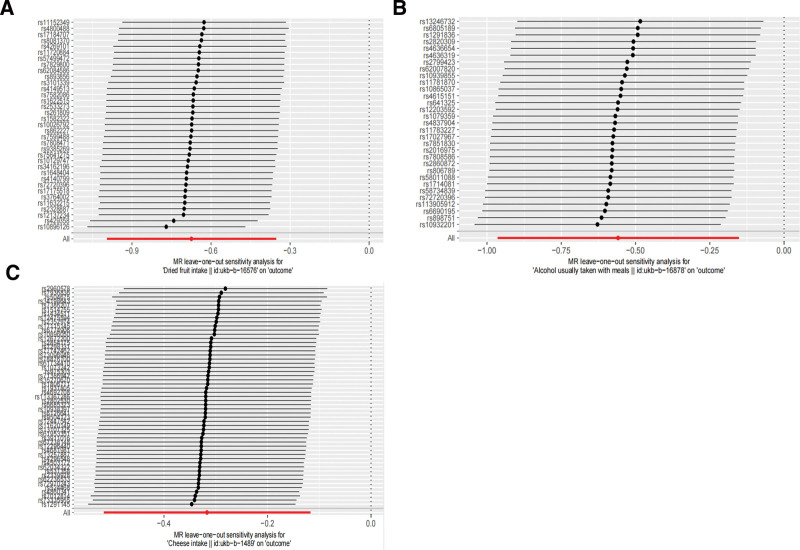
The experimental results were verified by using a leave-one-out sensitivity analysis. (A) Shows whether a particular SNP was removed to explore whether a specific SNP affects the overall outcome when using dried fruit intake as exposure. (B) Represents a leave-one-out sensitivity analysis of the alcohol usually taken with meals as exposure. (C) Represents a leave-one-out sensitivity analysis with cheese intake as exposure. The line represents the CI of that SNP, and the point on its line represents the OR value. We found that all lines in the 3 figures of (A), (B), and (C) are to the left of 0 and were slightly offset, indicating that the findings were less affected by a single SNP and that all 3 dietary habits showed a trend toward lower stroke risk. CI = confidence interval, OR = odds ratio, SNP = single-nucleotide polymorphism.

We then tested using MR-Egger intercept analysis and found that the *P*-values for MR-Egger were >0.05 (alcohol usually taken with meals [*P* = .11], dried fruit intake [*P* = .12], cheese intake [*P* = .54]), indicating no significant pleiotropy. Finally, we carried out a leave-one-out analysis, where the point represents OR and the segment represents CI, with OR and CI on the left of 0, and the overall offset was slight (Fig. 5). In Figure 5A, when we treated dried fruit intake as the exposure, we found that 2 SNPs (rs429358, rs10896126) had some impact on the overall offset, but this did not change the overall trend, and the degree of deviation was small, so the results can be considered stable. However, no significant shift was found when alcohol was usually taken with meals and cheese intake was exposed in Figure 5B and C, respectively. We can see that the 3 relatively significant eating habits were not affected by the results driven by a specific SNP as the exposure, demonstrating the robustness of our analysis.

## 4. Discussion

A preference for different diets may have some effect on stroke.^[6,14,38–40]^ Therefore, to investigate whether there was a direct causal relationship, we also performed an MR analysis. In this study, we analyzed the causality between 9 common dietary habits and stroke with MR to determine whether a particular dietary habit is a direct factor in triggering or alleviating stroke. With IVW, we found that there was a significant association between alcohol usually taken with meals, dried fruit intake, cheese intake, and stroke. These 3 dietary habits were associated with substantial reductions in stroke risk.

Although IVW is the most effective MR tool,^[28]^ this approach assumes that all genetic variants are valid IVs, whereas up to 50% of the weighted median is based on invalid IVs.^[30]^ Relying on a single method could lead to missed associations by being overly conservative when there is no pleiotropy or too many false positives by misspecifying the pleiotropic model.^[41]^ Therefore, the results obtained by the IVW method alone are not accurate.^[42]^ This result indicates that IVW alone cannot satisfy the judgment of positive results, requiring at least IVW and weighted median to meet both *P* < .05 to consider causality.^[43–45]^ From Table S2, Supplemental Digital Content, we found that the intake of only dried fruit met this condition (IVW: *P* = .00004, weighted median: *P* = .002), while both heterogeneity and pleiotropy tests found no problems. Therefore, we finally emphasized the association of food and alcohol usually taken with meals, cheese intake, and stroke, and the intake of dried fruit as an essential factor in the reduction of the risk of stroke.

The difference between our study and previous studies is also worth exploring. In earlier experiments, studies have shown that alcohol consumption provides cardiovascular protection mainly by improving insulin sensitivity and high-density lipoprotein cholesterol. Mild to moderate alcohol consumption is related to cardioprotective effects, and increasingly excessive alcohol consumption is proportional to outcomes.^[46]^ This suggests that the amount of alcohol varies and affects stroke. Even in the same group, there are still differences in the amount of alcohol that different people usually drink with meals. Genetic epidemiology suggests that alcohol consumption at all levels is associated with increased cardiovascular risk. Still, there are marked differences in risk at different consumption levels, including those accepted by current national guidelines.^[47]^ Regarding cheese intake, there are published MR articles similar to our study with the IVW method, indicating that cheese intake reduces the risk of ischemic stroke.^[48]^ For dried fruit intake, previous systematic evaluations and meta-analyzed prospective cohort studies have highlighted the benefits of different fruits for CVD.^[49]^ The macronutrients, micronutrients, and other bioactive compounds contained in dried fruits may synergistically regulate stroke risk through various mechanisms.^[50]^ Dried fruits are an excellent source of polyphenols and phenolic acids that reduce oxidative stress by scavenging or neutralizing oxidant species and enhancing endogenous antioxidant defenses against metabolic damage.^[51]^ Also, most dried fruits are good sources of dietary fiber and potassium, which are associated with lipid-lowering and vascular benefits, respectively, to improve vascular health and reduce the risk of stroke.^[52]^ Previous studies investigating the causal relationship between dried fruit intake and CVD have also found that dried fruit intake reduces the risk of total ischemic stroke and minor vessel stroke.^[13,53]^ In our study, the use of IVW has been associated with a reduction in the risk of stroke, which may slow the absorption of alcohol and reduce its adverse effects on the cardiovascular system. Moreover, the 9 genetic tools for dietary habits were derived from the IEU OpenGWAS project (https://gwas.mrcieu.ac.uk/). The definition of alcohol usually taken with meals comes from the original data of the IEU OpenGWAS project. The IEU OpenGWAS project did not provide detailed information on alcohol consumption. So in this analysis, it is a generalized genetic tendency IV. In the real world, the dietary habit of “alcohol usually taken with meals” encompasses significant heterogeneity, with substantial variations in alcohol types, frequency of consumption, and intake quantities.^[54–56]^ Therefore, the effect we obtained through MR should be interpreted as the average causal effect of this generalized behavioral pattern on stroke. Due to the inherently aggregated nature of the data, this study cannot differentiate between specific drinking patterns that may exhibit distinct biological effects. Future research requires more refined exposure data to elucidate more specific causal pathways. Regarding cheese intake, the IVW method shows that this exposure can reduce the risk of stroke, but we cannot draw a causal conclusion directly. Moreover, our selected sample size is larger than that of the previous study on ischemic stroke. It contains more data,^[48]^ which may be an essential reason for our inability to draw causal conclusions directly. Our study ultimately concluded that dried fruit intake reduced the risk of stroke, supported by 2 MR methods. Another study provided evidence of CVD benefits,^[53]^ which further demonstrated the reliability of our results.

To gain a deeper understanding of the findings of this study, we compared them with recent relevant literature.^[57,58]^ Guo et al shared overlapping dietary classification criteria with this study, both indicating that dried fruit intake can reduce the risk of stroke.^[57]^ Interestingly, Guo found that intake of oily fish and cheese could also reduce the risk of ischemic stroke. However, our study, which conducted causal analysis on a broader range of stroke types, did not demonstrate that oily fish intake could lower stroke risk. We speculate that this may be related to other stroke types, sample size, or other confounding factors. Furthermore, although the IVW analysis suggested a potential association between cheese consumption and reduced stroke risk, we did not draw direct causal conclusions after comprehensively considering the findings from other MR methods and factors such as sample size. Jareebi’s study adopted a relatively simplistic approach in dietary pattern selection, with a limited range of choices.^[58]^ Although it was found that fruit intake could reduce stroke risk, our study further investigated the comparison between fresh fruit consumption and dried fruit intake, unexpectedly revealing a strong correlation in dried fruit consumption. This conclusion was subsequently subjected to a detailed biological discussion. Therefore, our study possesses unique advantages.

MR provides a way to infer potential causal relationships between risk factors and outcomes from observational data, which is critical when randomized clinical trials are not feasible or unethical. As large genetic datasets become more accessible, MR has emerged as a powerful and user-friendly tool for investigating CVD risk factors.^[59]^ We also assessed the robustness and potential offset of the MR results using sensitivity analysis. Our study also used data sources with large sample sizes to improve the statistical efficacy of the study while reducing the random error, making the conclusion of causality more reliable. Moreover, our data were all selected from European populations, which avoids the shift of heterogeneity and enhances the validity of the results.

However, our study also had some deficiencies. The selection of the same population will also limit the results. The conclusions obtained cannot indicate that they can be applied to other populations. Moreover, we can only rely on the overall analysis, cannot conduct stratified analysis, and the stratified data volume cannot support practical statistical analysis, resulting in a significant error. In addition, our study was limited only to the food itself, and even if the MR design is adopted, the confounding factors cannot be excluded entirely. Environmental and other factors may also have an impact on the results.

## 5. Conclusion

In summary, we used 9 common dietary habits to investigate a possible causal link between diet and stroke. It was discovered that alcohol, usually taken with meals, dried fruit intake, and cheese intake were associated with stroke, and dried fruit intake was negatively correlated with stroke. This result indicates that dietary changes play a role in preventing and treating stroke, a role that should not be underestimated, and emphasizes the potential of diet to reduce stroke incidence.

## Acknowledgments

The authors thank all researchers who contributed to the OpenGWAS and GWAS Catalog databases for their willingness to make the data publicly available, and the mapping tools ChiPlot and Light-Scholar for providing a platform to help complete the visualization of critical data.

## Author contributions

**Data curation:** Fei He, Na Li, Zhen Wang, Yixuan Li.

**Validation:** Fei He, Jie Yang.

**Visualization:** Fei He, Shangling Xie.

**Writing – original draft:** Fei He.

**Writing – review & editing:** Si Yuan.




