# Supplementary material for: Dietary habits and stroke: A Mendelian randomization analysis
Source: Medicine (Baltimore). 2026 May 8;105(19):e48588. doi: 10.1097/MD.0000000000048588 (PMC13166574; doi:10.1097/MD.0000000000048588)
Supplement: Supplementary file 1 [file medi-105-e48588-s001.docx]

Salt added to food

Bread intake

Cereal intake

Cheese intake

Fresh fruit intake

Dried fruit intake

Oily fish intake

Non-oily fish intake

Supplementary Table 1: Detailed data on the SNPs selected from nine common dietary habits.
