# Supplementary material for: Dietary habits and stroke: A Mendelian randomization analysis
Source: Medicine (Baltimore). 2026 May 8;105(19):e48588. doi: 10.1097/MD.0000000000048588 (PMC13166574; doi:10.1097/MD.0000000000048588)
Supplement: Supplementary file 2 [file medi-105-e48588-s002.docx]

Supplementary Table 2: Detailed MR calculation results of three dietary habits, namely alcohol usually taken with meals, dried fruit intake and cheese intake, obtained by inverse variance weighted (IVW), weighted median, weighted mode, simple mode, and MR-Egger.
